# Diagnostic, Prognostic, and Mechanistic Biomarkers of Diabetes Mellitus-Associated Cognitive Decline

**DOI:** 10.3390/ijms23116144

**Published:** 2022-05-30

**Authors:** Hanan Ehtewish, Abdelilah Arredouani, Omar El-Agnaf

**Affiliations:** 1 Division of Biological and Biomedical Sciences (BBS), College of Health & Life Sciences (CHLS), Hamad Bin Khalifa University (HBKU), Doha 34110, Qatar; hehtewish@hbku.edu.qa; 2 Diabetes Research Center, Qatar Biomedical Research Institute (QBRI), Hamad Bin Khalifa University (HBKU), Doha 34110, Qatar; 3 Neurological Disorders Research Center, Qatar Biomedical Research Institute (QBRI), Hamad Bin Khalifa University (HBKU), Doha 34110, Qatar

**Keywords:** Alzheimer’s disease, cognitive dysfunction, dementia, mild cognitive impairment, type2 diabetes mellitus

## Abstract

Cognitive dysfunctions such as mild cognitive impairment (MCI), Alzheimer’s disease (AD), and other forms of dementia are recognized as common comorbidities of type 2 diabetes mellitus (T2DM). Currently, there are no disease-modifying therapies or definitive clinical diagnostic and prognostic tools for dementia, and the mechanisms underpinning the link between T2DM and cognitive dysfunction remain equivocal. Some of the suggested pathophysiological mechanisms underlying cognitive decline in diabetes patients include hyperglycemia, insulin resistance and altered insulin signaling, neuroinflammation, cerebral microvascular injury, and buildup of cerebral amyloid and tau proteins. Given the skyrocketing global rates of diabetes and neurodegenerative disorders, there is an urgent need to discover novel biomarkers relevant to the co-morbidity of both conditions to guide future diagnostic approaches. This review aims to provide a comprehensive background of the potential risk factors, the identified biomarkers of diabetes-related cognitive decrements, and the underlying processes of diabetes-associated cognitive dysfunction. Aging, poor glycemic control, hypoglycemia and hyperglycemic episodes, depression, and vascular complications are associated with increased risk of dementia. Conclusive research studies that have attempted to find specific biomarkers are limited. However, the most frequent considerations in such investigations are related to C reactive protein, tau protein, brain-derived neurotrophic factor, advanced glycation end products, glycosylated hemoglobin, and adipokines.

## 1. Introduction

Dementia is a neurodegenerative condition initiated many years before the appearance of overt clinical deficits of memory, thinking, reasoning, and behavioral changes. Dementia is the most progressive stage of cognitive dysfunction, with impairment of multiple cognitive domains that interfere with daily life activities. The 2015 World Alzheimer’s report estimated that 46.8 million people worldwide were living with dementia in 2015, likely reaching 74.7 million by 2030 [1]. Alzheimer’s disease (AD) is the most common cause of dementia, affecting 60–70% of individuals aged 65 years or older [2]. As with dementia, the risk for developing diabetes mellitus (DM) increases with age. According to the International Diabetes Federation (IDF), 463 million people (9.3%) had diabetes in 2019, and this figure is expected to increase to 700 million (10.9%) by 2045. Type 2 diabetes mellitus (T2DM) accounts for 90% of all diabetes cases [3]. Cognitive impairment and dementia (including AD) are increasingly recognized as common complications and comorbidities of T1 and T2DM [4]. Thus, diabetes is associated with a 1.25–1.9-fold higher risk for cognitive dysfunction [5], including AD, with a relative risk (RR) of 1.53, and vascular dementia (VD), with RR of 2.27 [5]. The domains of information processing speed, attention and concentration, executive functioning, and working memory are most affected among people with diabetes [6]. Mild cognitive impairment (MCI) is a heterogeneous condition, described as an intermediate stage between the expected decline in cognitive function due to normal aging and dementia, but with minimal impact on everyday activities. Typically, MCI affects memory, language, thinking, and judgment ability. According to the American Academy of Neurology, the prevalence of MCI increases with age. It is estimated that 7% of individuals aged 60–64 years are affected, while approximately 25% of people aged 80–84 may develop cognitive impairment [7]. Although a meta-analysis study showed that 14–55% of MCI cases return to a normal cognition state, 15% of individuals older than 65 with MCI progress to dementia [7]. Diabetes was associated with a higher risk of MCI with a hazard ratio of 1.14, and the risk of conversion from mild cognitive impairment to dementia is 1.53 times higher among people with diabetes [5].

**Figure 1:**
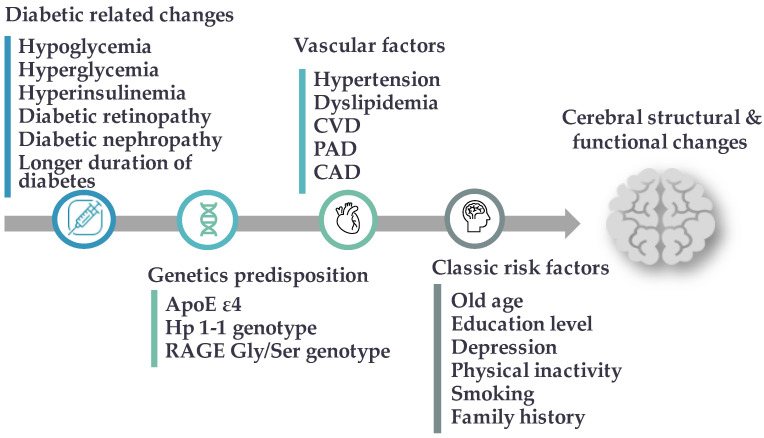
Potential risk factors for cognitive impairment and dementia in type 2 diabetes mellitus. Abbreviation s: CVD: cerebral vascular disease, PAD: peripheral arterial disease, CAD: coronary artery disease, Hp: Haptoglobin protein, RAGE: Receptor for advanced glycation end products.

The established relationship between dementia and T2DM is reciprocal, with cognitive impairment increasing the risk of severe hypoglycemia episodes, cardiovascular diseases, stroke, and early death [8]. Several diabetes-related complications, including glucose and insulin imbalances as well as the microvascular and macrovascular comorbidities, were found to significantly predispose patients to the progression of MCI and its conversion to dementia [9]. Moreover, several risk factors for cognitive dysfunction in diabetes were determined, including cardiovascular risk factors such as hypertension and dyslipidemia, depression, age, diabetes duration, and educational status of patients with diabetes [10] (Figure 1). However, some discrepancies in risk estimations for cognitive dysfunction have been noted, and the existing evidence needs to be evaluated further. A better understanding of the relationship between diabetes and cognitive impairment may help identify patients at high risk of developing cognitive decline and guide healthcare teams to provide appropriate preventive strategies [10].

Hyperglycemia and the subsequent production of reactive oxygen species are the proposed triggers involved in the progression of T2DM chronic complications [11]. However, the mechanisms underlying the cerebral changes in diabetes are still not well understood. Studies have suggested neurodegeneration as a possible mechanism whereby diabetes may be linked to a higher risk of AD. However, AD pathology, including the accumulation of amyloid-β and abnormal tau phosphorylation, is still being investigated as a mechanism of DM-related cognitive impairment [9]. Additionally, advanced glycation end products (AGEs), vascular endothelial dysfunction, dysglycemia, insulin dysregulation, and neuroinflammation may be involved in the pathogenesis of diabetes-associated cognitive decline [9].

The global rise in the prevalence of T2DM and its associated cognitive disorders, and the lack of specific biochemical indicators and effective strategies to treat dementia or delay cognitive decline, all contribute to the increased demand for early diagnosis biomarkers. The potential application of these biomarkers in early detection and the possibility of therapy to monitor diabetes-related cognitive dysfunction is of paramount clinical importance. Conclusive investigations into specific biomarkers for diabetes-associated cognitive dysfunctions are limited and inconsistent. Besides the early changes in cerebral metabolism and brain structures that can be detected using brain imaging technology platforms [4], the most promising molecules identified so far are related to glucose metabolism, inflammation, neurodegeneration, vascular damage, and oxidative stress. Some of these molecules include C reactive protein (CRP) [12], interleukin-6 (IL-6) [13], leptin and interleukin-1 (IL-1 β) [14], homeostasis model assessment of the insulin resistance (HOMA-IR) score along with glycosylated hemoglobin (HbA1c) [15], advanced glycation end products (AGEs) [16], tau protein [17], glycogen synthase kinase 3β (GSK-3β) [18], brain-derived neurotrophic factor (BDNF) [19] and microRNAs [20]. In the following sections, we aim to evaluate the contributory risk factors and the potential pathophysiological mechanisms of cognitive disorders in diabetes, and to discuss the potential biomarkers related to the cognitive decline in T2DM patients.

## 2. Risk Factors for Cognitive Dysfunction in Diabetes Mellitus

### 2.1. Hypoglycemia

Diabetes mellitus and its related complications, such as hypoglycemia, were recognized as a risk factor for dementia. In streptozotocin-induced diabetic rats, severe hypoglycemia episodes were associated with significant neuronal damage in both the cortex and hippocampus [21]. Several studies also found that hypoglycemic people have a doubled risk of dementia progression compared to those who have not had hypoglycemic incidents [22,23,24] (Table 1). Moreover, the risk increased with increased numbers of hypoglycemic episodes [24,25]. On the other hand, diabetes patients with dementia and an inability to self-manage had an almost threefold higher risk of having severe hypoglycemic events [22,26]. This two-way association was supported by the Edinburgh Type 2 Diabetes Study, which reported that a history of hypoglycemia was associated with steeper cognitive decline, and cognitive disability was associated with a greater risk of being affected by severe hypoglycemia [27]. Moreover, a population-based retrospective cohort study involving 5,966 patients who experienced at least one episode of hypoglycemia revealed that hypoglycemia was associated with a higher risk of all-cause dementia, AD, and VD, with hazard ratios of 1.26 and 1.28, respectively [28]. A recent study showed that 23% of cases with hypoglycemia developed all types of dementia, especially AD, in comparison to only 7% among those who did not experience hypoglycemia. The risk increased from 1.5 for individuals with one episode of hypoglycemia to 1.8 for those who had two or more episodes [29].

### 2.2. Hypertension

The link between hypertension and the risk of cognitive dysfunction was identified early, because untreated long-term hypertension is associated with structural artery wall alterations that cause chronic cerebral hypoperfusion [42,43]. Other studies, however, failed to find a similar association [44,45]. It is hypothesized that the age of onset of hypertension, the chronicity of hypertension, and the antihypertensive medication utilized are all critical factors in determining the risk of cognitive dysfunction [46,47,48,49]. People with T2DM are commonly diagnosed with hypertension [50], which is linked with an increased risk of diabetes-associated cognitive decrements [51] (Table 2), while high diastolic blood pressure was demonstrated to be comorbid with T2DM and was shown to be a predictive factor for AD development [52]. In addition, an observational six-year follow-up study reported that severe systolic hypertension significantly elevated the risk of dementia and its subtypes, including VD, when it co-existed with diabetes [53]. Another longitudinal study of aging reported hypertension as a risk factor for cognitive decline only when it occurred with diabetes [51]. Middle-aged and older adults’ structural and diffusion MRI brain scans showed that hypertension and diabetes were independently associated with grey and white matter macrostructural and microstructural changes [54]. However, other studies revealed no significant trend related to the incidence of dementia in patients who have comorbid diabetes with hypertension [53].

### 2.3. Diabetic Retinal Microangiopathy

Influenced by the anatomical and pathophysiological similarities of the retinal and cerebral microvasculature, several studies investigated the potential for providing non-invasive predictors of cognitive abnormalities by assessing retinal vascular changes. The risk of cognitive impairments, including dementia, was observed in patients with T2DM who had manifestations of diabetic micro-vascular abnormalities, such as diabetic retinopathy [30,60] (Table 1). A longitudinal study involving 29,961 patients with T2DM showed that subjects with diabetic retinal abnormality had a 42% greater risk of dementia [31]. Furthermore, a systematic review and meta-analysis revealed that 12 out of 15 studies associated retinopathy with a higher risk of MCI progression to dementia [10] However, the findings were inconclusive, as other observational studies reported that the presence of retinopathy or neuropathy was not significantly associated with either cognitive decline or brain structural abnormalities over four years [32]. On the other hand, another study showed that retinopathy was associated with severe cerebral atrophy but not with accelerated cognitive decline [61].

### 2.4. Diabetic Renal Function Impairment

Chronic renal disease has been reported to increase the risk of cognitive decline independent of diabetes and other vascular risk factors [33] (Table 1). The concurrence of diabetes and impaired kidney function was reported to increase the risk of cognitive impairment considerably [34]. Renal dysfunction indicators, urinary albumin to creatinine ratios (UACR), and elevated cystatin C levels were significantly and independently associated with a decline in cognitive function [35,36,37,38]. Even though no association with the estimated glomerular filtration rate (eGFR) was found in some investigations [35], other studies reported that a decline in eGFR was associated with poor performance in cognitive tests [38,39]. Moreover, low eGFR and high urinary albumin excretion rate (UAER) were considered independent risk factors for diabetes-associated cognitive dysfunction [40]. Additionally, diabetes patients with MCI were reported to have increased UACR and cystatin C levels and decreased eGFR. However, after further adjustment of metabolic and cardiovascular risk factors, only high serum cystatin C was associated with an increased risk of MCI, with an OR of 1.42 [41].

### 2.5. Macrovascular Disease

In addition to the abovementioned diabetic microangiopathy complications, diabetic patients with manifestations of other microvascular and macrovascular abnormalities have a higher risk of cognitive dysfunction [42]. Coronary artery disease, cerebrovascular disease, and peripheral arterial disease were shown to be independent vascular risk factors and predictors of cognitive dysfunction development in people with diabetes [52,56,57] (Table 2). As a result of atherosclerosis, thromboembolic stroke is the most well-known contributor to the development of diabetes-related cognitive dysfunction [4]. The risk was noted to be approximately twice as high in those with previous events of cerebrovascular disease (with a hazard ratio (HR) 2.03; 95% CI 1.88–2.19) [57]. Albai et al. also supported the link, reporting that a history of stroke and cardiovascular diseases was associated with a higher risk of dementia in diabetes patients [58].

Moreover, the Edinburgh Type 2 Diabetes Study found that with prior stroke, markers of subclinical macrovascular stress, including higher plasma levels of the natriuretic peptide NT-proBNP, and increased carotid intima-media thickness were associated with a steeper cognitive decline in patients who were diagnosed with T2DM [59]. Other studies, however, showed that high levels of the natriuretic peptide are associated with cognitive impairment and dementia in the general population as well. Therefore, definitive evidence of a correlation in the case of T2DM has not yet been provided [62]. Peripheral arterial disease was linked to a higher risk of dementia, with an HR of 1.47, and was suggested as an independent predictor of dementia, with odds ratios of 5.35 (95% CI 2.08–13.72) [52]. Multiple studies also revealed that appropriate management of diabetes patients resulted in fewer vascular comorbidities and lowered the risk of dementia progression [63].

### 2.6. Dyslipidemia

Dyslipidemia refers to unhealthy high levels of triglycerides and/or low-density lipoprotein (LDL) and low levels of high-density lipoprotein (HDL) [64]. Dyslipidemia is a common cardiovascular risk factor strongly associated with atherosclerotic lesions and ischemic stroke [42]. T2DM is also commonly known to be a risk factor for atherosclerotic lesions and ischemic stroke, with likelihoods of 21% and 18%-22%, respectively [65]. In the general population, it was expected that 20% of the post-stroke population would develop dementia, and the risk of dementia increases by 3% per year after a stroke [66]. A systematic review of Mendelian randomization studies reported that higher total cholesterol and LDL were linked to higher AD risks; in contrast, higher HDL was associated with a lower incidence of AD [67]. In the case of T2DM, a few studies showed a link between dyslipidemia and the risk of cognitive dysfunction. However, the evidence for dyslipidemia as a risk factor is largely inconclusive.

### 2.7. Genetic Factors

Haptoglobin protein (Hp) is an extracellular secreted chaperone that plays a critical protective role against improper extracellular protein aggregation. The Hp-β protein is significantly downregulated in AD, 1.9 times lower than in MCI, and 1.7 times lower in AD than control subjects [68]. Furthermore, the Hp-β chain was shown to be 3.4 times more oxidized in AD than in healthy controls and 2.7 times more oxidized in AD than in MCI cases, suggesting the potential function of Hp-β in the progression of MCI to dementia [68]. Carrying the haptoglobin-1-1 (Hp-1-1) genotype was found to be significantly associated with poor cognitive performance [69], and a decrease in right hippocampal volume in T2D patients with poor glycemic control was also observed [70]. Diabetes-related micro- and macro-vascular problems, including diabetic cognitive impairment, have been linked to AGEs [11]. The receptor for advanced glycation end products (*RAGE*) Gly/Ser genotype was reported to be associated with a higher risk of AD, independently of the *ApoE* E4 gene [71]. However, Wang et al. [16] reported that, despite an elevated serum AGE level in MCI diabetics, *RAGE* genotypes were not associated with cognitive deterioration in diabetics. In addition, in T1DM, *ApoE* E4 genotypes were not related to cognitive dysfunction in subjects followed up for 18 years [72]. The E4 *ApoE* allele was shown to be the most common among T2DM patients with MCI, and its expression was related to inferior cognitive performance in T2DM subjects with MCI than in E2 and E3 carriers [73]. Concerning the *ApoE* allele E4, the genotypes E3/4 and E4/4 had the highest frequencies among T2DM patients with AD (71.15% and 18.59%, respectively) compared to the control group (81.03% and 6.90%, respectively) [74]. The carriers of the E3/4 genotype had higher triglycerides and LDL-C and lower HDL-C levels, suggesting that the E3/4 genotype is a potential risk factor for AD in T2DM [74] Although *ApoE* E3/4 or E4/4 alleles were most common in dementia patients with T2DM, the association was significant when the E3/4 genotype was combined with hemochromatosis-HFE gene mutations (H63D and C282Y) in females and with prior cerebrovascular events in males [75].

### 2.8. Other Risk Factors

The essential factor that causes neurodegenerative diseases is aging [76]. An increase in the age of diabetes patients is considered a risk predictor for dementia progress, the risk being 50% higher in over-75 than in 65–75 year old patients [10]. Furthermore, greater severity and longer duration of T2DM significantly increased the risk of diabetes-related cognitive decline [52,77]. Higher educational attainment correlates with reduced risk of cognitive dysfunction, including AD [78]. Additionally, according to a 2020 Lancet Commission report on dementia prevention, intervention, and care, 40% of dementia cases were related to multiple modifiable risk factors, including smoking, obesity, hearing loss, hypertension, diabetes, physical inactivity, and depression [63].

Depression was also suggested to play a role in cognitive decline in T2DM patients. Hence, in a prospective study, Katon et al. [79] showed that the risk of dementia in individuals with comorbid depression and DM was twice as high as it was in those who only had diabetes. According to a national population-based cohort study, T2DM and depression were independent risk factors for dementia progression with a hazard ratio of 1.83 among depressive patients, 1.20 among subjects with diabetes, and 2.17 among those with both conditions when compared with healthy subjects [80]. However, Trento et al. [81] reported that despite an increase in depression prevalence in diabetic patients (20.9%), no significant association of depression with cognitive decline was observed compared to the general population.

## 3. Mechanisms of Cognitive Dysfunction in Diabetes Mellitus

The precise underlying pathogenic mechanisms of cognitive decline in T2DM have not yet been thoroughly defined and are almost certainly complex, with multiple interacting variables. Along with the causes underlying AD pathology, various endocrinological, metabolic, and vascular abnormalities are DM-related, such as hyperinsulinemia, insulin resistance, and poor glycemic control; these may hasten the deterioration of cognitive abilities.

### 3.1. Dysglycemia (Hypo and Hyperglycemia)

**Figure 2:**
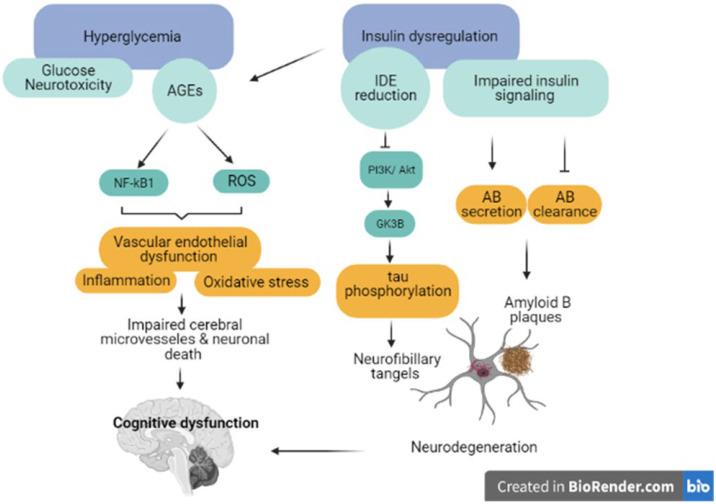
The potential mechanisms linking type 2 diabetes mellitus-related changes with cognitive decline and dementia progression. Abbreviations: AGEs: advanced glycation end products, ROS: reactive oxygen species, IDE: insulin degradation enzymes, AB: amyloid β.

T2DM characterized by hyperglycemia is closely connected to decreased cognitive performance, but the exact mechanism involved is still unknown. However, it is known that hyperglycemia’s acute and chronic consequences are both involved. Acute hyperglycemia causes osmotic changes in cerebral neurons by altering the regional cerebral blood flow, which results in oxidative stress and subsequent neuronal damage [82]. On the other hand, chronic hyperglycemia mediates neuronal damage through the formation of AGEs which, in turn, leads to the formation of reactive oxygen species (ROS) and the production of pro-inflammatory cytokines, resulting in microvascular changes and systemic inflammation [83]. Evidence of the contributions of recurrent episodes of acute hypoglycemia to cognitive function worsening in patients with DM has been reported [27,28]. However, the mechanisms of hypoglycemia-induced neuronal damage are not well known [84]. Severe hypoglycemia is associated with diffuse neuronal death that may result from excitotoxicity, oxidative stress, polymerase-1 activation, postsynaptic zinc accumulation, and the mitochondrial release of pro-apoptotic molecules [42] (Figure 2).

### 3.2. Insulin Resistance and Hyperinsulinemia

Insulin resistance (IR) and hyperinsulinemia were widely identified as risk factors for cognitive impairment and dementia, including AD, independently of diabetes [85,86]. However, the mechanisms through which IR may cause neuronal damage in T2DM have not yet been identified. Insulin has multiple functions in the brain, including food intake and energy homeostasis modulation [83]. Additionally, insulin receptors are distributed abundantly throughout the hippocampus, entorhinal cortex, and frontal lobes, whose functions are involved in memory, attention, and execution [87]. This observation suggests that insulin may play a significant role in cognition by regulating cerebrocortical activity and brain metabolism and, most likely, by controlling the production of the neurotransmitter acetylcholine [88,89]. Alterations in insulin signaling pathways, phosphorylation of Insulin Receptor Substrate 1, and altered insulin-like growth factor-1 signaling were observed in the AD brain, suggesting the potential role of insulin in cognitive dysfunction pathogenesis [84,90].

Additionally, IR and hyperinsulinemia were linked to AD pathological features, β-amyloid neurotic plaques (NPs), and intracellular neurofibrillary tangles (NFTs) formed of hyperphosphorylated tau protein [91]. IR and hyperinsulinemia are suggested to be associated with an increase in the secretion and decrease of the extracellular clearance of Aβ as a result of reduced insulin-degrading enzyme system (IDE) synthesis [88]. Furthermore, the impaired insulin-PI3K-Akt signaling observed in the AD brain enhances glycogen synthase kinase-3β, increasing the tau protein’s phosphorylation [92,93]. Additionally, insulin was linked to cognitive dysfunction through the mediation of vascular damage due to the vasoactive effect of insulin and the production of AGEs [83] (Figure 2).

### 3.3. Neuro-Inflammation

Proinflammatory cytokines such as tumor necrosis factor (TNF), IL-1, IL-2, and IL-6 are overexpressed in diabetes and Alzheimer’s disease patients’ brains [94], indicating the role of inflammation in neuronal damage, likely through the downregulation of the pro-inflammatory microglial function [95]. Through glucose neurotoxicity and a defective insulin signaling system, hyperglycemia, insulin dysregulation, and oxidative stress were connected to neuronal apoptosis, neuroinflammation, and the development of neurodegeneration in diabetes [96,97]. This activates transcription factors, AGE/RAGE, polyol, and protein kinase C pathways [96]. Additionally, oxidative stress, hyperinsulinemia, and hyperglycemia play significant roles in the activation of NF-κB, which, in turn, modulates apoptosis and ROS production and regulates the expression of TNF and interleukins, the inflammatory cascade enhancers in the brain cells [98]. Furthermore, inflammation was linked to disruption of the blood-brain barrier, which exposes the neurons to toxic substances and induces abnormal neuronal function [9].

### 3.4. Glucocorticoid Excess and Perturbed HPA Axis Function

Dysregulation of the hypothalamic–pituitary–adrenal (HPA) axis was demonstrated in patients with T2DM [97,99]. High cortisol levels were associated with metabolic changes and increased risk of diabetes-related vascular complications such as ischemic heart disease, retinopathy, neuropathy, and nephropathy [82]. It has been proposed that, through the destructive effect of excess glucocorticoids on the hippocampal and entorhinal neuronal functions [100], increased glucocorticoid levels, either exogenous or endogenous, play a role in the pathogenesis of cognitive decline and dementia, as well as in psychological issues in T2DM patients [101,102]. Furthermore, elevated cortisol was associated with hippocampal atrophy and impairment in humans’ hippocampus-dependent learning and memory tasks [103]. In animal models, cognitive dysfunction was related to the impaired hippocampal function mediated by glucocorticoid effects on neurogenesis and synaptic plasticity [104]. Several studies proposed that the altered activity of the HPA axis contributes to T2DM-related cognitive dysfunction. HPA axis impairment was associated with decreased declarative memory performance and reduced hippocampal and prefrontal volumes in T2DM patients [99,105]. In support of this, the Edinburgh Type 2 Diabetes Study demonstrated the relationship of high fasting cortisol levels with more significant cognitive decline, which mainly affected working and processing speeds [106]. Further detailed studies are required to better understand whether disruptions in the HPA axis in T2DM lead to hippocampal damage and contribute to the pathology of cognitive impairments. This will have the potential for therapeutic manipulations to improve cognitive function.

### 3.5. The Role of Vascular Pathology

Small vessel diseases, including atherosclerosis and cerebral amyloid angiopathy, are common cerebrovascular diseases connected to cognitive impairment and dementia [107]. White matter changes, including white matter hyperintensities (WMHs), are small vessel neuroimaging features linked to cognitive dysfunction and neurodegenerative disorders, including AD [108]. In a longitudinal study, Yan-Li Wang et al. [109] reported that higher WMHs were significantly associated with cognitive dysfunction, and thus increased the risk of dementia. In the case of T2DM, increased WMH lesions also appeared on MRI; however, the link was inconsistent despite this finding. The contribution of white matter integrity changes to the progression of diabetes-associated cognitive decline is controversial [110].

Nonetheless, Mankovsky et al. [111,112] reported increased WMH volumes associated with lower information processing speed among diabetes subjects with less controlled blood glucose. Assessment of the structural properties of the cerebral white matter via diffusion tensor imaging consistently showed a decrease in fractional anisotropy or an increase in mean diffusivity, which was negatively correlated with cognitive performance in T2DM patients [9,113]. Many of these white matter abnormalities were detected in normally cognitive diabetic, pre-diabetic, and insulin-resistant individuals, suggesting an early commencement of T2DM’s effect on brain structure and function [110].

Furthermore, MRI images showed that T2DM is associated with cerebral vascular injuries, mainly manifested as lacunar infarcts, but the relationship between diabetes and the risk of cerebral microbleeds was inconsistent [9]. Cerebral microvascular function investigations revealed that T2DM patients have decreased global or regional cerebral blood flow; however, this has not been linked to cognitive ability [112]. A new whole-brain arterial spin-labeling MRI method study found that regional decreases in cerebral blood flow were related to worse memory and executive function/processing speeds in older individuals with T2DM [114,115]. T2DM has also been associated with diminished cerebral vasoreactivity [113], which can exacerbate cognitive deterioration in diabetes patients [4]. Vascular endothelial dysfunction related to diabetes is mainly linked to the accumulation of AGEs, toxic lipids, and protein aggregates in the small vessels [9]. This protein deposition enhances ROS production while lowering vasodilatory substances, eventually impairing cerebral blood flow, increasing capillary permeability, decreasing cerebral glucose intake, and promoting cerebral neuronal damage [9].

### 3.6. Neurodegeneration

Brain imaging studies report consistent associations between T2DM and progressive brain atrophy in areas affected by AD, including the hippocampus, suggesting that T2DM induces AD-related pathology [89]. As discussed above, insulin resistance and hyperinsulinemia increase the levels of intracellular and extracellular Aβ, resulting in Aβ accumulation and abnormal tau phosphorylation via the insulin-signaling activation of the GSK-3b cascade [92]. However, the cerebral accumulation of Aβ as a mechanism of DM-related cognitive impairment is still under investigation. Additional cerebral protein aggregates are suggested to be involved in the neurodegenerative changes in diabetes [4,9]. For instance, pancreatic β-cell hormone Amylin was connected to T2DM pathogenesis. Abnormal secretion of Amylin was found to coincide with disturbance in insulin secretion in pre-diabetes and late phases of T2DM. It was also identified as one of the major components of pancreatic islet amyloid deposits in T2DM patients [116,117]. It was additionally reported that the over-expression of human amylin induces the accumulation of amylin in rat brains, leading to neurological deficits that are mediated by a neuroinflammatory response [118].

Increased amylin secretion was proposed to have a novel role in the mechanism of brain dysfunction in diabetic patients [119]. A retrospective autopsy-based study reported cytoplasmic depositions of tau and Aβ proteins in pancreatic β cells, and amylin aggregation in the hippocampus of subjects with AD and subjects with normal cognition but a history of T2DM; interestingly, the amylin was found to be colocalized with tau or Aβ in pancreatic and brain tissue [120]. Increased amylin accumulation, elevated blood glucose levels, insulin resistance, and Aβ deposition in the hippocampus are linked to a depletion in social cognition and learning ability in human islet amyloid polypeptide (hIAPP) transgenic mice fed with a high-fat diet [121].

Altogether, the data suggest that the over-expression of amylin, in addition to the Aβ and tau tangles, plays a crucial role in both T2DM and AD pathologies [122]. However, more research is needed to better understand the exact role of amylin in the development of neurodegenerative diseases such as AD and assess the feasibility of employing amylin as a biomarker for neuronal damage in diabetes patients.

## 4. Biomarkers of Cognitive Dysfunctions in Type 2 Diabetes Mellitus

### 4.1. Neurodegeneration-Related Biomarkers

#### 4.1.1. β-Amyloid (Aβ) and Hyper-Phosphorylated Tau

The primary neuropathophysiological hallmarks of AD were recognized to be β-amyloid extracellular neurotic plaques (NPs) and hyper-phosphorylated tau intracellular neurofibrillary tangles (NFTs) [123,124,125].

Although the incidences of all-cause dementia, including AD and VD, were found to be twice as high in diabetes as in non-diabetes subjects [126], some studies have assumed that the development of cognitive impairment in elderly diabetes individuals is primarily related to neurovascular dysfunction, rather than to the primary AD pathologies. No evidence to demonstrate differences in Aβ and hyper-phosphorylated tau accumulations among diabetes patients has been detected. Previously, Heitner and colleagues [127] ruled out diabetes as a risk factor for AD because there was no significant difference in Alzheimer-type pathology parameters between diabetes and control participants [127]. This hypothesis was supported by a study of 268 diabetes participants, with or without AD, which revealed that diabetes subjects had significantly fewer neuropathological features of AD (NPs and NFTs) in the cerebral cortex and the hippocampus than non-diabetes subjects [128]. Additionally, an immunostaining retrospective postmortem study reported that the *ApoE* E4 allele was the one that most influenced the extent of AD pathology (many NPs and NFTs), but not hyperglycemia [129]. On the other hand, Moran et al. [130] reported a strong association between diabetes and lower bilateral frontal and parietal cortical thickness and an increase in CSF phosphorylated tau (p-tau). Still, neither brain Aβ load nor CSF Aβ levels were related to diabetes. That was subsequently confirmed by a study that indicated an increase in the rate of functional activity decline among patients with both diabetes and one of the AD risk variables, either genetic susceptibility, such as carrying the *ApoE* E4 allele, or CSF alterations of total tau and p-tau, but not CSF Aβ [131]. Additionally, DM was found to be associated with alteration only in CSF total tau level, but not in p-tau or Aβ in *ApoE* E4 carriers diagnosed with MCI or early dementia [17].

Several Positron Emission Tomography (PET) studies used radioligands to visualize Aβ plaque deposition and confirmed the absence of a link between T2DM and cerebral amyloid deposition [4]. A recent PET amyloid and tau accumulation tracers study reported that 52% of patients with diabetes-associated cognitive decline were tau positive, indicating tauopathy-related dementia, while only 29% had an Alzheimer-type pathology [132]. Similarly, another study showed that approximately 30% of those clinically diagnosed with AD among the DM participants were tau positive. However, Aβ negativity was significantly associated with metabolic abnormalities of T2DM, greater impairment of frontal lobe function, and a slower progression of cognitive decline [133]. Another PET imaging study indicated that cerebral cortical Aβ was considerably lower in people with T2DM than subjects without T2DM, consistent with previous findings [134] The same study discovered that the AD biomarker CSF Aβ was higher in the T2DM group than in the non-diabetes group [134].

In support of the link between T2DM and neurodegeneration, it was observed that long-term treatment with the most commonly used oral hypoglycemic drug, Metformin, was associated with reduced diabetes-related cognitive decline when compared to other anti-diabetes drugs [135,136,137]. The exact function of Metformin in reducing neurodegenerative changes is not well understood. Still, studies suggest the involvement of Metformin in different pathways, including in reducing Aβ, through decreasing beta-secretase 1 (BACE1) protein activity [138]. However, more studies are needed to investigate the association between the CSF and plasma levels of AD-type biomarkers and diabetes-associated cognitive dysfunction.

#### 4.1.2. PI3K and GSK-3B

Phosphoinositide 3-Kinase is a signal transmitting protein that participates in glucose metabolism, and its dysregulation is associated with high blood glucose levels [124]. T2DM development in rats was linked to a significant decrease in insulin signaling pathway proteins such as PI3K, phosphorylated-Akt, mTOR, and GSK3 [139]. Furthermore, a PI3K deficiency is linked to an increase in AD pathology and hyperphosphorylation and tau protein deposition via an increase in GSK-3 activity [140]. In addition, dramatic decreases in the levels and activities of the insulin/phosphoinositide 3-kinase/protein kinase B (insulin–PI3K–AKT) signaling pathway in AD and T2DM subjects were reported [141]. These observations suggest the involvement of PI3K in the pathophysiology of diabetes and AD. In support of this, Xu et al. [142] observed that adiponectin significantly alleviates cognitive impairments in T2DM rats by activating the PI3K/Akt/GSK-3 pathway and decreasing tau hyperphosphorylation.

GSK-3 is thought to be an interface between diabetes and AD due to its role in the PI3K/Akt signaling pathway and the phosphorylation of tau protein. GSK-3 dysregulation was associated with the development of insulin deficiency and insulin resistance, as well as aberrant tau phosphorylation [140]. Higher peripheral circulating GSK-3β was reported to correlate negatively with cognitive scores in diabetes patients who had been diagnosed with MCI, and it has been suggested as a diagnostic marker for mild cognitive impairment in T2DM patients [18].

### 4.2. Inflammatory Markers

Elevated acute-phase protein levels have emerged as an early indication of the development of T2DM [141]. Increasing levels of chronic subclinical inflammatory markers, such as C-reactive protein (CRP) and interleukin-6 (IL-6), are thought to be involved in the development of T2DM via insulin resistance onset and insulin secretion disruption [143,144,145]. An association between inflammation and accelerated decline in cognitive function in diabetes patients has been reported [146]. In contrast, only a few studies investigated links between inflammation biomarkers and cognitive decrements among diabetes subjects, with contradictory results [13,14,147,148,149]. For instance, a recent study that included 37 T2DM participants involved in the CAROLINA^®^ trial revealed no association between cognitive deterioration and the markers of inflammation CRP, IL-6, and tumor necrosis factor-α (TNF-α) [147]. However, a cross-sectional study (Edinburgh T2DM Study) demonstrated that elevated plasma IL-6 and TNF-α, but not CRP, are significantly associated with poor cognitive performance [13].

Nevertheless, high levels of CRP were found to be associated with lower cerebral vasoreactivity and vasodilation, which, in turn, were linked to a decline in executive function and activities of daily living in T2DM participants [148]. Furthermore, serum CRP concentrations were significantly higher in MCI patients with T2DM, positively correlated with HbA1c levels, and negatively correlated with cognitive assessment scores. Among diabetes patients with no MCI, the opposite was true, suggesting that an increased CRP level in the circulation can be an early indicator of MCI development in diabetes patients [12]. In another study, Gorska-Ciebiada and colleagues [149] reported significant elevations of the inflammatory biomarkers CRP, IL-6, and TNF-α in T2DM patients who had been diagnosed with MCI, with or without a depressive mood. Serum concentrations of the proinflammatory cytokines IL-1 β were significantly higher in elderly diabetes patients with MCI, but only a weak positive correlation with the cognitive assessment was observed [14]. Additionally, plasma CRP, IL-6, and lipoprotein-associated phospholipase A2 levels were significantly higher in diabetes patients with MCI compared to T2DM controls without MCI [150,151]. Given these inconsistent findings, more studies are required to investigate the potential of using inflammatory molecules as predictive and diagnostic biomarkers of diabetes-associated cognitive dysfunction.

### 4.3. Oxidative Stress Markers (Advanced Glycation End Products)

Advanced glycation end products (AGEs) are a complex and heterogeneous group of compounds produced by the non-enzymatic modification of tissue proteins by physiologically reduced sugars [4]. AGEs accumulate naturally with age, and their deposition is enhanced by hyperglycemia and oxidative stress, as seen in T2DM patients [152,153,154,155]. Diabetes has been linked to an increase in the buildup of AGEs [152]. When compared to the brains of people with AD alone, immunohistochemical labeling of human post-mortem brains of those with AD and diabetes revealed an increased number of receptors for AGEs (RAGE) and higher AGE levels [154], suggesting that an oxidative stress mechanism that is promoted by AGEs may underpin the development and severe progression of AD pathology in diabetes patients. On the other hand, Chen et al. [156] reported higher levels of serum AGEs and lower levels of endogenous secretory RAGE in T2DM patients with MCI.

Furthermore, a cross-sectional investigation of 167 diabetes participants revealed that serum AGE levels were raised in MCI subjects, and this rise was inversely connected with global cognitive performance and associated with a 72% increase in MCI risk in T2DM patients; however, serum soluble RAGE concentration decreased in diabetes patients with MCI, and an increase in the RAGE level was associated with a 54% reduction in disease risk [16]. This finding proposes that RAGE protects against cognitive impairment in diabetes patients, partly by blocking AGEs–RAGE interactions. However, these findings are inconsistent with results from previous studies where serum RAGE and AGEs levels were significantly high and inversely correlated with cognitive test scores in MIC patients with T2DM [12]. Given this inconsistency, evidence from longitudinal studies is required to elucidate the potential of serum levels of AGE and RAGE as predictive biomarkers for diabetes-associated cognitive decline.

### 4.4. Neurotrophic Factors—BDNF

The brain-derived neurotrophic factor (BDNF) is an essential member of the neurotrophin family of growth factors for maintaining learning and memory functions by regulating cell survival, synaptic plasticity, and proliferation, and reducing neuroinflammation in the central nervous system [157]. Diabetes patients’ blood levels of BDNF were significantly lower than healthy controls [158], and this depletion was mediated by diabetes-induced chronic hyperglycemia and AGE accumulation [159]. BDNF serum levels were also found to be significantly reduced in T2DM patients and those with all kinds of diabetes-induced dementia, including VD and AD [19]. Furthermore, a significant decrease in serum BDNF levels was associated with impaired cognitive function and was positively correlated with delayed memory and attention in patients with T2DM [158,160]. Additionally, it was found that blood BDNF levels in T2DM patients with MCI were significantly lower than in patients without MCI, and it was estimated that the risk of MCI would decrease by 6% with each one-unit increase in BDNF [161]. Furthermore, in a study involving 155 Chinese patients with diabetic retinopathy and/or nephropathy, serum BDNF levels were nearly half as low as among those who had no complications, thus suggesting BDNF as a potential independent indicator of diabetes complications [162]. Ren et al. [163] found that plasma BDNF concentrations were considerably lower in T2DM participants older than 60 years. However, there was no significant association between plasma BDNF and cognitive performance, thus ruling out BDNF as a biomarker for cognitive dysfunction in T2DM.

The frequency of BDNF C-270T polymorphism was found to be higher among AD patients than cognitively normal controls; this allele was reported to increase the risk of AD independently of the *ApoE* E4 allele with OR of 3.13 and 3.8 among German and Japanese populations, respectively [164,165]. Although BDNF levels were not significantly different among three BDNF genotype subgroups in diabetes patients and healthy subjects, there was a significant positive correlation between BDNF levels and delayed memory score in BDNF Val66Met homozygote carriers, suggesting the involvement of this polymorphism in the development of cognitive impairment [166]. Interestingly, in animal models, the over-expression of BDNF was shown to improve glucose metabolism and alleviate insulin resistance [167] by suppressing the hyperglycemia-induced neuroinflammation in the hippocampus of type 1 diabetes mice through blocking the HMGB1/RAGE/NF-κB signaling pathway [168].

These data indicate the importance of investigating the role of BDNF in the pathophysiology of diabetes-associated cognitive deterioration and the potential applications of BDNF as a diagnostic and therapeutic tool in diabetes-associated cognitive dysfunction.

### 4.5. Adipokines

Adipokines are adipose tissue-released mediators that include leptin, TNF-α, IL-6, adiponectin, adipsin, heparin-binding epidermal growth factor (HB-EGF), and vascular endothelial growth factor (VEGF). They are involved in the pathogenesis of many diseases, such as obesity-related cognitive impairment, by promoting angiogenesis, inflammation, cell proliferation, and insulin resistance [169]. High energy intake has been linked to brain structure and function abnormality, cognitive deficits, and dementia [169,170]. The adipocytokines, leptin, and adiponectin were related to insulin resistance-mediated cognitive dysfunction [171]. Elevated serum leptin levels were associated with poorer cognitive performance, mainly within the cognitive domains of mental flexibility and executive function, among older men with T2DM, but not in women [172]. Serum levels of leptin and IL-1 β were found to be significantly increased, and adiponectin decreased, in T2DM patients with MCI, suggesting that higher levels of leptin and IL-1 β, as well as lower levels of adiponectin, could be diagnostic biomarkers of MCI risk in elderly diabetes patients [14].

Additionally, circulating low adiponectin levels were associated with lower hippocampus volume [173], lower grey matter volume, and decreased cerebral glucose metabolism in the parietotemporal regions in T2DM patients [174]. In contrast, another study found that increased plasma adiponectin levels in T2DM women were associated with an increase in the risk of developing all-cause dementia, including AD [175]. The serum and CSF levels of the serine protease adipokine adipsin were higher in patients with T2DM [176] and in T2DM patients with MCI [177]. Adipsin levels in the blood were significantly and positively connected with HbA1c but adversely correlated with cognitive test scores [177]. These results suggest high plasma levels of adipsin are an independent risk factor for MCI.

Altogether, these findings show the link between the adipokines and cognition. Further extensive cohort studies are needed to confirm the roles of adipokines in diabetes-related cognitive impairment pathogenesis and investigate the potential of adiponectin and leptin as diagnostic markers of cognitive dysfunction.

### 4.6. Diabetes-Related Biochemical Biomarkers

#### 4.6.1. Hyperinsulinemia and Insulin Resistance

Hyperinsulinemia is an early pathophysiological response to insulin resistance in T2DM progression [62]. An association between hyperinsulinemia and cognitive deterioration in T2DM patients was observed, in which elevated levels of the homeostasis model assessment of the insulin resistance (HOMA-IR) score, along with glycosylated hemoglobin (HbA1c), were associated with a significant decline in the cognitive performance of diabetes subjects [15,178]. Additionally, a higher plasma insulin level and IR were associated with decreased attention/executive function and memory impairment in older patients with T2DM [179]. By comparison, Geijselaers et al. [180] showed that the results of fasting insulin, C-peptide, and HOMA-IR were not related to the cognitive performance of 641 T2DM individuals who were not receiving insulin treatment [180]. However, another recent study showed that, along with AGE levels, insulin resistance was higher among diabetes participants and was negatively correlated with cognitive functions. In contrast, glucose-dependent insulinotropic peptide and plasma glucagon-like peptide-1 were positively correlated with cognitive performance [181]. Moreover, fasting insulin and HOMA-IR were higher in diabetes participants with MCI than in those with normal cognition [182]. A cross-sectional study including 157 T2DM patients reported that C-peptide levels were lower and positively correlated with cognition scores in patients with MCI, suggesting reduced C-peptide level as an independent risk factor for MCI among diabetes subjects [183].

An argument has been put forward regarding the association of plasma insulin with cognition in people with diabetes, stating that the progression of cognitive dysfunction may be due to diabetes and the associated changes in brain metabolites and brain structures, rather than the effect of hyperinsulinemia itself [62]. However, some studies report a negative correlation between cognitive performance and fasting insulin and HOMA-IR in non-diabetes participants with hyperinsulinemia and insulin resistance (IR) [184]. Moreover, hyperglycemia and IR were associated with executive function and memory impairment, respectively, in non-diabetes participants with MCI [185]. Higher HOMA-IR was also associated with poorer verbal fluency in young and middle-aged females [186], and increased the risk of cognitive decline by 46% in women [187]. Additionally, a systematic review and meta-analysis showed that exogenous hyperinsulinemia increases the risk of dementia in diabetes patients compared to non-diabetes subjects and diabetes individuals not receiving insulin infusion, with RR of 1.83 and 1.36, respectively [5]. This data proposes that high plasma insulin may be an early marker of an increased risk of cognitive decline in diabetes and the healthy elderly.

#### 4.6.2. Hemoglobin A1C (HbA1c) and Hyperglycemia

Multiple studies have investigated the link between chronic hyperglycemia and diabetes-related cognitive disorders. However, the results were inconsistent. A cross-sectional analysis of 302 T2DM participants revealed no significant differences in fasting glucose and HbA1c in diabetes patients with or without dementia [52] or in T2DM patients with MCI [183]. Additionally, HbA1c levels were stable and not correlated with cognitive decline over six years of T2DM patient observations [179]. These results were supported by a systematic meta-analysis of five RCTs, which showed that the rate of cognitive deterioration is unrelated to the stability of HbA1c levels in T2DM patients [188]. Manschot et al. [189] reported a modest association between cognitive performance, the level of HbA1c, and the diabetes duration. Another study noted that HbA1c levels in T2DM cases were significantly associated with impaired information processing speed and abstract reasoning domains [61]. Furthermore, analysis of approximately 3,000 T2DM subjects from the ACCORD-MIND trial reported inverse correlations between chronic hyperglycemia measured by HbA1c levels and and cognitive performance [190]. Interestingly, the relationship of HbA1c to cognitive impairment was reported independently of diabetes; a prospective community-based cohort study showed that elevated levels of fasting glucose and HbA1c were associated with an increased risk of dementia in those with and without diabetes [191]. A recent meta-analysis reported that high HbA1c values were associated with increases in the risk of dementia and MCI with pooled RR of 1.27 and 1.95, respectively, when not considering HbA1c as a continuous variable [5]. Another group of studies suggests that mid-life diabetes is a risk factor for late-life cognitive impairment. Participants with high HbA1c levels had the worst cognitive performance [192,193]. Additionally, diabetes subjects with HbA1c ≥ 7% and a midlife glucose peak had a 0.4 times greater drop in cognitive function [194].

### 4.7. MicroRNAs

MicroRNAs (miRNA) are short (21 to 23 nucleotides), single-stranded, non-coding RNA molecules, mainly functioning in RNA silencing and the post-transcriptional regulation of gene expression via base-pairing to the complementary sequences in their target mRNAs [195]. Recently, many miRNA dysregulations were reported in metabolic tissues, such as adipose, liver, skeletal muscle, and the insulin-producing pancreatic β cells, in both human and animal models, and were suggested to contribute to the pathogenesis of many diseases [196,197]. miRNAs were demonstrated to participate in glucose metabolism, insulin secretion, fat metabolism, adipocyte differentiation, energy homeostasis, and inflammation [197]. Insulin resistance is one of the multifactorial mechanisms in DM etiology, and various miRNAs, such as miR-320, miR-128a, miR-29, miR-384-5p, miR-143, miR-383, miR-33a/b and miR-133a/b, are reported to target the genes of the main proteins in the insulin signaling pathway and to play a crucial role in developing insulin resistance in adipose tissues [198]. Reduced miR-221 and miR-28-3p and increased circulating levels of miR-486-5p, miR-486-3p, miR-142-3p, miR-130b, and miR-423-5p were observed in children with obesity, and this alteration was significantly related to insulin resistance [199]. Plasma levels of miR-21 were significantly lower in the obese population with and without DM and negatively correlated with obesity-associated metabolic changes such as hyperinsulinemia [200]. In pre-diabetes subjects, miR-126 and miR-146a were reduced and subjects could be distinguished from healthy controls based on the circulating levels of miR-146a, miR-126, miR-30d, and miR-148a [201]. Moving to diabetes, plasma levels of 13 miRNAs were dysregulated in subjects with DM; five of these (miR-15a, miR-126, miR-320, miR-223, miR-28-3p) correctly distinguished cases with DM [202]. Additionally, serum levels of circulating miRNAs (miR- 486, miR-146b and miR-15b) were consistently upregulated and discriminated patients with T2DM [203].

A study demonstrated that the expression levels of miR-661, miR-571, miR-770-5p, miR-892b, and miR-1303 were significantly higher in T2DM patients with microvascular complications such as nephropathy, neuropathy, and retinopathy in comparison to those without complications [204]. Low serum levels of miRNAs related to the amyloid precursor protein (APP) proteolysis, miR-20a, -27a, and -103a, were associated with significantly low cognition scores in patients who had not been diagnosed with dementia among the Japanese population [205]. A recent systematic review of microRNA expression in AD showed seven dysregulated miRNAs in the brain of AD patients, including five downregulated miRNAs (miR-16-5p, miR-107, miR-132-3p, miR-181a/c/d-5p, and miR-212-3p) and two upregulated (miR-34-5p and miR-125a/b-5p). However, the results were contradictory and inconsistent for most of these miRNAs among studies regarding circulating tissues, both in CSF and blood [206]. miRNA-132 was downregulated in the brain tissue of late AD stage patients and suggested to contribute to AD pathogenesis via affecting cholinergic function and inducing tau phosphorylation [207,208]. Serum levels of miR-132 were upregulated in MCI patients, correlated significantly with the cognitive assessment score [209], and correctly separated MCI patients from age-matched healthy controls [210]. A recent study involving 163 participants reported that the plasma levels of miR-132 were significantly higher in T2DM patients with MCI compared to normally cognitive subjects [20].

miR-223 is associated with neuroinflammation through modulating the nuclear factor kappa B (NF-κB) pathway [211]. Serum miR-223 levels were reduced in MCI patients, and further significantly downregulated and positively correlated with cognitive score in individuals with AD [212,213,214]. miR-146a is another neuroinflammatory mediator and is expected to be involved in the pathogenesis of some neurological disorders, including AD [215]. miR-146a is significantly upregulated in AD brain tissue [216]. However, the changes in the expression of circulating levels of miR-146a were contradictory among studies [206]. Maffioletti et al. [217] observed no alteration in miR-146a expression in the plasma of AD subjects compared to the healthy controls; however, negative correlations with cognitive scores and severity of the cognitive deterioration were reported. By contrast, Ansari et al. reported significantly higher levels of miR-146a and miR-181a in the plasma of MCI patients who converted to AD [218]. Several micro-RNAs are involved in T2DM, diabetes complications, and neurodegeneration in the literature. Future studies to explore the role of these micro-RNAs in diabetes-associated cognitive dysfunction is required, giving the potential of new diagnostic and therapeutic tools for T2DM complications.

### 4.8. Homocysteine

Homocysteine is a non-proteinogenic α-amino acid, strongly linked to vascular endothelial cytotoxicity and neuronal injury. Several clinical observations have demonstrated that an elevated plasma homocysteine level is associated with various cognitive dysfunction conditions such as AD, VD, cognitive impairment, and stroke [124,219,220]. This correlation was observed in patients with cognitive dysfunction and individuals with a declining cognitive performance [221]. Elevated levels of homocysteine were also shown to increase the risk of T2DM in a randomized meta-analysis of 4011 diabetes cases [222]. Additionally, hyperhomocysteinemia was observed in patients who had been diagnosed with diabetic macrovascular and microvascular complications [223,224,225,226]. Only a few studies have investigated the connection between the plasma levels of homocysteine and diabetes-associated cognitive impairments, and the results remain equivocal. Damanik et al. [227] could not find any significant difference in the level of homocysteine between T2DM patients with and without MCI. However, Tian and his colleagues reported that increased plasma levels of homocysteine were negatively correlated with cognitive scores and significantly associated with MCI in 285 T2DM patients [228]. The exact pathophysiological mechanisms underlying such complications are not well understood. However, it is believed that homocysteine promotes oxidative stress via methylation disturbance that promotes calcium influx, amyloid and tau protein accumulation, neuronal death, and neurodegeneration [219].

Identifying biomarkers of diabetes-related cognitive disorders is crucial for understanding the condition’s biological characteristics and as a detection tool for diagnosis and early screening. However, the current findings showed huge inconsistency; the biomarkers discussed in this review are summarized in Table 3.

## 5. Conclusions

This review provides an overview of pathophysiological links between T2DM and dementia. Despite the strong and consistent epidemiological evidence for an increased risk of cognitive dysfunctions in T2DM patients, the definitive mechanisms underlying the link between T2DM and dementia pathogenesis remain elusive. Current mechanistic studies provide multiple pathophysiological leads, including the metabolic disturbances in T2DM, cerebral insulin resistance, accumulation of glycation end products, vascular endothelial dysfunction, neurodegeneration, and inflammation. However, many critical questions remain unanswered. In addition to the traditional dementia risk factors such as age, educational status, cardiovascular risk factors, and genetic predisposition, recent studies have identified other diabetes-specific risk factors, including prolonged duration of diabetes, inadequate glycemic control, and diabetes-related microvascular complications. Due to the heterogeneity of the available evidence, more studies are needed to elucidate the pathogenesis of the disease and T2DM-related dementia risk factors required to optimize adequate management measures and determine definitively mechanistic and prognostic markers. Despite the rising aging population and the prevalence of age-related diseases such as T2DM and diabetes complications, including cognitive impairment and dementia, there are still no well-established biomarkers for diabetes-associated cognitive decrements. There is great potential for biomarker development in this field; biomarkers related to diabetes metabolic changes, inflammatory response, oxidative stress, and neurodegeneration were identified but showed inconsistencies. Future studies using a well-characterized, longitudinal, large population cohort are in great demand to validate the current findings and to identify sensitive and specific biomarkers for early detection of cognitive decline in diabetes patients.

## Figures and Tables

**Table 1:** Studies showing the associations between type 2 diabetes mellitus complications, cognitive decline, and dementia progression.

Study	Design	Participants’ Number and Mean Age	Cognitive Measure	Risk Factor Measures	Association with Cognition
[22]	Prospective population-based follow-up study	783 diabetes patients; mean age of 74.0 years	Modified MMSE scores	Hypoglycemia was determined from hospital records	Participants with a hypoglycemic event had a twofold increased risk for developing dementia.
[23]	Follow-up study	15,404 diabetes patients; mean age of 64.2 years	ICD-9-CM codes	ICD-9-CM codes	The incidence rate of dementia was higher in diabetic subjects with hypoglycemic episodes.
[24]	Longitudinal cohort study	16,667 diabetes patients; mean age of 65 years	ICD- 9-CM code	ICD-9-CM codes	A history of severe hypoglycemic episodes was associated with a greater risk of dementia
[25]	Retrospective longitudinal cohort study	53,055 diabetes patients; aged >65 years	Defined based on CPRD data	Clinical Practice Research Datalink (CPRD)	The risk of dementia increased with an increasing number of hypoglycemia episodes.
[27]	Prospective ET2D study	831 diabetes patients; aged 60–75 years	MMSE score	Participants self- completed questionnaires.	Both history of hypoglycemia and incident hypoglycemia were associated with poor initial cognitive ability and accelerated cognitive decline.
[28]	Population-based retrospective observational study	5,966 diabetes patients; mean age 75.82 years	ICD-10 codes	ICD-10 codes	Patients with hypoglycemia had a higher risk for dementia, including AD and VD
[30]	Cross-sectional analyses of a longitudinal study	1,862 diabetes patients; mean age of 62.3 years	MMSE score; DSST scores	Eye examinations and fundus photography	Diabetic retinopathy was associated with lower grey matter volume and future cognitive decline.
[31]	Longitudinal cohort study	29,961 diabetes patients; aged 60 years	ICD 9 codes	ICD 9 codes	Those with diabetic retinal disease had a 42% increased risk of incident dementia.
[32]	Longitudinal study	68 T2DM patients; mean age of 65.6 years	Neuropsychological assessment of major cognitive domains.	Albuminuria; Single-field retinal photographs; Toronto Clinical Neuropathy Scoring System	Albuminuria predicted accelerated cognitive decline in patients with T2DM, while retinopathy and neuropathy did not.
[33]	Cross-sectional analyses of prospective observational study	3591 diabetes patients; mean age of 58.2 years	Modified MMSE	eGFR	The prevalence of cognitive impairment was higher among those with lower eGFR, independent of traditional vascular risk factors.
[34]	Cross-sectional study	1,358 diabetes patients; aged ≥60 years	MMSE scores	eGFRcys	Comorbid diabetes with impaired kidney function was associated with a higher risk of cognitive impairment in older adults.
[35]	Randomized controlled trial	2,968 diabetes patients; mean age of the entire cohort was 62.5 years	MMSE scores; RAVLT; DSST; Stroop test	eGFR; ACR; cystatin C	Kidney dysfunction was associated with cognitive decline in diabetes patients at high risk of cardiovascular diseases.
[36]	Randomized controlled trial	2977 diabetes patients; mean age of 62.5-65.8 years	MMSE scores; RAVLT; DSST; Stroop test	Albuminuria; eGFR	Albuminuria was associated with a decline in cognitive function in relatively young individuals with diabetes with unimpaired eGFR.
[37]	Cross-sectional hospital-based study	79 diabetic patients; mean age of 76.0 years	MMSE scores; DSST; Stroop test	Albuminuria; eGFR; SBIs; WMLs	Albuminuria and low eGFR were associated with a decline in DSST scores (frontal lobe dysfunction), but not with MMSE or Word Recall scores.
[38]	Cross-sectional analyses of longitudinal studies	67 diabetes patients; mean age of 74.6 years	MMSE; DSST; Stroop test; Word recall scores	Albuminuria; eGFR; ACR	ACR was strongly and independently associated with changes in word recall scores; eGFR decline was associated with a greater decrease in MMSE and DSS scores.
[39]	Longitudinal study	649 diabetes patients; mean age of 70 years	Thirteen neurocognitive tests	eGFR	Low eGFR was associated with reduced executive function performance.
[40]	Retrospective study	216 diabetes patients	MMSE; MoCA	eGFR; UAER	MMSE and MoCA scores were negatively correlated with the UAER and positively correlated with the eGFR.
[41]	Cross-sectional relation	357 diabetes patients, mean age of 66.58 years	MoCA	Serum Cystatin C; eGFR; UAER	Only elevated serum Cystatin C was associated with increased risk of cognitive impairment in diabetic patients, independent of conventional risk factors.

Abbreviations: ET2DS (The Edinburgh Type 2 Diabetes Study), DSST (Digit Symbol Substitution Test), eGFR (estimated glomerular rate), eGFRcys (cystatin C-based estimated glomerular filtration rate), ACR (albumin/creatinine ratio), RAVLT (Rey Auditory Verbal Learning Test), SBIs (silent brain infarcts), WMLs (white matter lesions), UAER (urinary albumin excretion rate), MoCA (Montreal Cognitive Assessment).

**Table 2:** Studies showing the relationships between vascular risk factors and cognitive function in patients with diabetes.

Study	Design	Number	Cognitive Measure	Association with Cognition
[51]	Population-based longitudinal study on aging	258 participants (mean age = 83 years)	MMSE score	Higher cognitive decline among individuals with comorbid diabetes and hypertension.
[55]	Community-based, longitudinal study on aging and dementia	1301 participants (mean age = 81.5 years)	DSM-III-R criteria	Increased risk for dementia among DM patients with severe systolic hypertension or heart disease.
[53]	Retrospective cohort study	51,580 patients aged between 20 and 99 years	ICD-9-CM	The dementia risk in patients with hypertension and hyperlipidemia did not significantly increase in patients with DM.
[52]	Cross-sectional study	302 participants aged 75.7 ± 4.6 years	(MMSE) score IQCODE	Diastolic blood pressure and peripheral arterial disease were independent predictors for dementia.
[56]	Longitudinal cohorts T2DM patients aged 60 +	29,961 patients aged above 60 years	ICD-9-CM	Microvascular disease, diabetic foot, cerebrovascular disease, and cardiovascular disease most strongly predicted dementia.
[57]	Population-based matched cohort study	225,045 with and 668,070 without DM participants mean age = 73 years	ICD-9 -CM	The risk of dementia was most significant among seniors with diabetes, a prior cerebrovascular disease, and peripheral vascular disease.
[58]		207 participants (mean age = 57.49)	MMSE score	Stroke events, cardiovascular disease, and higher LDLc levels were significant risk factors for MCI conversion to dementia.
[59]	prospective Edinburgh Type 2 Diabetes Study (ET2DS).	831 participants (aged 60–75 years)	MMSE score	Stroke and subclinical cardiac stress markers were associated with cognitive decline in older patients with T2DM.

Abbreviations: MMSE score (Mini-Mental State Examination), DSM-III-R (The Diagnostic and Statistical Manual of Mental Disorders, revised third edition criteria), ICD-9-CM (International Classification of Diseases, Ninth Revision, Clinical Modification), IQCODE (Informant Questionnaire for Cognitive Decline in the Elderly), ET2DS (The Edinburgh Type 2 Diabetes Study).

**Table 3:** Summary of the biomarkers of diabetes-related cognitive dysfunction discussed in this review.

Biomarkers	The Effect/Function	Biomarker Alteration	Link with T2DM	The Association with Diabetic Cognation Dysfunction	Ref
**Plasma CRP/(IL-6)/(TNF-α)**	Acute-Phase Proteins, a marker of a chronic subclinical inflammation	Increased	Insulin resistance initiation and insulin secretion disturbance	Inflammatory biomarkers not related to cognitive performance. Only IL-6 and TNF-α, but not CRP, correlated with poor cognitive performance.	[13,147]
				Associated with a decline in executive function and daily living activities and positively correlated with MCI in T2DM patients.	[148,150]
**β-amyloid (Aβ)/phosphorylated tau**	Neuropathological features of AD	Increased	Impaired insulin signaling associated with tau hyperphosphorylation, Aβ accumulation	The cerebral cortex and the hippocampus NPs and NFTs were not correlated with DM.	[128,134]
				Increase in CSF phosphorylated tau, but neither brain Ab load nor CSF Ab42 associated with AD in people with diabetes.	[131]
**PI3K**	Signal transmitting proteins that participate in glucose hemostasis.	Decreased	Increased blood glucose levels and a significant decrease in insulin–PI3K–AKT signaling pathway.	Positively correlated with T2DM and the deposition of tau protein.	[140]
**BDNF**	Neurotrophin growth factor regulating cell survival, synaptic plasticity, reduction of neuroinflammation in CNS.	Decreased	Ameliorates glucose metabolism and alleviate insulin resistance.	No significant association between plasma BDNF and cognitive performance in T2DM was reported.	[163]
				Low BDNF serum levels positively correlated with MCI, VD, and AD in T2DM.	[19,157]
**AGEs**	Product of a nonenzymatic series of chemical reactions between glucose and proteins.	Increased	AGE formation is accelerated by hyperglycemia and oxidative stress in DM.	Serum RAGE and AGEs levels were significantly high and inversely correlated with cognitive test scores in MIC patients with T2DM	[12]
				Higher levels of serum AGE and lower levels of RAGE in type 2 diabetes patients were inversely correlated with global cognitive function.	[11,153]
**Adiponectin**	Adipose tissue secreted mediators	Decreased	Maintain the metabolism of glucose and lipids, sensitizes the body to insulin, and anti-inflammatory molecule	Increased plasma adiponectin was associated with an increased risk of development of all-cause dementia in diabetic women.	[175]
				Adiponectin was decreased in type 2 diabetic patients with MCI, lower hippocampus and grey matter volumes, and decreased cerebral glucose metabolism.	[14,173]
**HOMA-IR/Fasting insulin, C-peptide**	A peptide hormone that maintains normal blood glucose levels.	Increased	Insulin resistance and type 2 diabetes mellites progression	Fasting insulin, C-peptide, HOMA2-IR is not related to cognitive performance.	[180]
				High insulin levels and dropped C-peptide negatively correlated with cognitive function.	[179,183]
**HbA1c**	Glycated hemoglobin reflects average plasma glucose over the previous 8 to 12 weeks	Increased	Biomarker for diagnosis and prognosis of T2DM.	FPG and HbA1c were not associated with cognitive decline and MCI.	[52]
				High HbA1c levels in type 2 diabetes cases were inversely significantly associated with worse cognitive performance.	[190]
**miRNA**	Regulates glucose and fat metabolism, insulin secretion, adipocyte differentiation, energy homeostasis, and inflammation.	Dysregulated	Targets the genes in insulin signaling pathway proteins and IR progression.	Low plasma levels of miR-20a, -27a, and -103a are associated with low cognition scores.	[205]
				Plasma levels of miR-132 were higher in MCI patients with T2DM.	[20]
**Amylin**	Glycemic regulation via regulating energy intake and body weight.	Increased	Coincides with insulin secretion disturbance in pre-diabetes, and induction of pancreatic β-cells toxicity.	Hippocampal Amylin protein aggregate correlated with T2DM with/without AD.	[122]

Abbreviations: CRP (C reactive protein), IL-6 (Interleukin 6), TNF-α (Tumor necrosis factor-α), PI3K (Phosphoinositide 3-Kinase), BDNF (Brain-derived neurotrophic factor), AGEs (Advanced glycation end products), HOMA-IR (the homeostasis model assessment of the insulin resistance), HbA1c (Hemoglobin A1C), miRNA (MicroRNAs).

## Data Availability

Not applicable.

## Abbreviations

AD	Alzheimer’s disease
AGEs	Advanced glycation end products
BDNF	Brain-derived neurotrophic factor
CRP	C reactive protein
HbA1c	Glycosylated hemoglobin
HOMA-IR	Insulin resistance score
Hp	Haptoglobin protein
HPA	Hypothalamic-pituitary-adrenal
IL-1	Interleukin-1
IL-6	Interleukin-6
MCI	Mild cognitive impairment
miRNA	microRNAs
NFTs	Neurofibrillary tangles
NPs	β-amyloid neurotic plaques
PET	Positron Emission Tomography
PI3K	Phosphoinositide 3-Kinase
RAGE	Receptor of advanced glycation end products
ROS	Reactive oxygen species
T2DM	Type 2 diabetes mellitus
TNF-α	Tumor necrosis factor-α
VD	Vascular dementia
WMHs	White matter hyperintensities
